# Stability of a ZEISS LISA tri 839MP multifocal IOL with a missing haptic segment in a high myopic patient: A 3-year follow-up case report

**DOI:** 10.1097/MD.0000000000042042

**Published:** 2025-04-11

**Authors:** Jae-Ho Shin, Jong Won Lee, Jong Ho Lee, Hui Jae Lee

**Affiliations:** a Department of Ophthalmology, College of Medicine, Kyung Hee University Hospital at Gangdong, Seoul, Korea; b Department of Ophthalmology, Seoul Ire eye clinic, Seoul, Korea.

**Keywords:** cataract surgery, high myopia, long-term follow-up, missing haptic, multifocal IOL stability

## Abstract

**Rationale:**

The purpose of this study was to present the long-term outcomes of a monobloc multifocal intraocular lens (IOL) (ZEISS LISA tri 839MP) with a missing haptic segment in a patient with high myopia.

**Patient concerns:**

A 51-year-old female with high myopia presented for cataract surgery.

**Diagnoses:**

The patient was diagnosed with cataracts in both eyes, with high myopia as a pre-existing condition.

**Interventions:**

Phacoemulsification with implantation of a ZEISS LISA tri 839MP multifocal IOL was performed. During surgery, 1 haptic was torn and removed. The remaining 3 haptics of the plate-haptic-designed IOL were positioned in the capsular bag.

**Outcomes:**

Postoperative uncorrected distance visual acuity improved to 20/20 on the first day, with stable intraocular pressure and good centration of the IOL. Over the 3-year follow-up period, the IOL remained well-centered with no significant decentration, and the patient maintained stable visual acuity of 20/20.

**Lessons:**

The ZEISS LISA tri 839MP multifocal IOL demonstrated long-term stability and favorable visual outcomes despite the missing haptic segment, suggesting that conservative management may be effective in similar cases.

## 1. Introduction

Intraocular lens (IOL) implantation is a crucial step in cataract surgery, particularly challenging in patients with high myopia due to increased risks of zonular instability associated with elongated axial length and thinner scleral tissue, complicating both the surgical process and the postoperative course).^[1,2]^ Complications during IOL insertion, such as haptic tearing, can further affect outcomes, potentially leading to IOL decentration or dislocation.^[3]^

**Figure 1.**
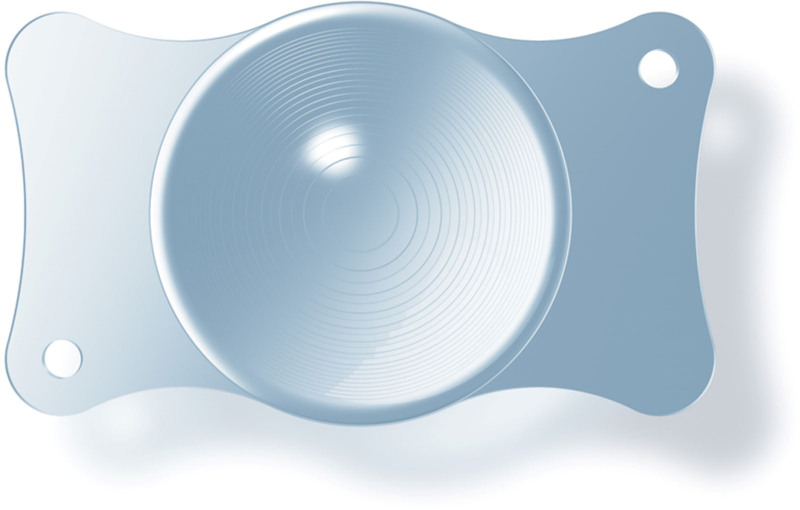
The ZEISS LISA tri 839MP multifocal intraocular lens (IOL) features a monobloc design with plate haptics, rather than a traditional C-loop haptic design. This figure is used with the permission of ZEISS.

Multifocal IOLs, such as the ZEISS LISA tri 839MP, are designed to provide clear vision at various distances but require proper centration and stability within the capsular bag to optimize visual outcomes. The lens is a monobloc design with plate haptics, not a C-loop haptic design.^[4,5]^ (Fig. 1). In cases where 1 or more haptics are damaged or missing, IOL stability may be jeopardized, particularly in eyes with preexisting conditions like high myopia.^[6]^

Current knowledge indicates that decentration of a trifocal intraocular lens (IOL) can significantly affect visual outcomes, particularly contrast sensitivity and intermediate vision. Decentration, even as little as 0.4 mm, has been shown to negatively impact visual quality, with the degree of effect depending on the specific design of the IOL.^[7]^ Furlan et al^[8]^ demonstrated that a new trifocal IOL design, incorporating a Devil diffractive lens, showed increased resistance to decentration, leading to improved performance, particularly in intermediate distances. Furthermore, studies by Megiddo-Barnir et al^[9]^ and Alió et al^[10]^ have reinforced the finding that decentration of diffractive trifocal IOLs leads to decreased visual outcomes, such as reduced contrast sensitivity and less optimal intermediate vision, highlighting the importance of IOL centration for preserving visual quality. Lin et al^[11]^ also noted that decentration of trifocal lenses could lead to significant patient dissatisfaction due to blurred vision, especially under lower lighting conditions. Moreover, Santhiago et al^[12]^ provided further evidence that optical performance in terms of sharpness and clarity worsens as decentration increases, stressing the importance of precise IOL positioning to maximize visual outcomes. These findings underscore the need for careful consideration of IOL centration during cataract surgery to minimize the negative effects of decentration on visual performance.

This case report details the successful management of a ZEISS LISA tri 839MP multifocal IOL with a missing haptic segment in a high myopic patient and documents IOL stability and visual outcomes over a 3-year follow-up.

## 2. Case report

**Figure 2.**
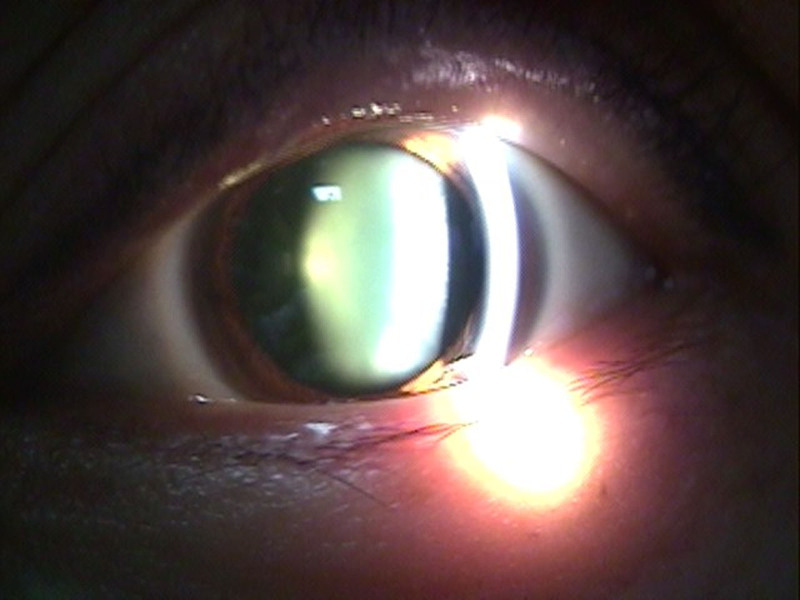
Slit-lamp microscopic examination shows findings consistent with cataract (LOCUS NC 3, NO3). This figure is used with the permission of ZEISS.

**Figure 3.**
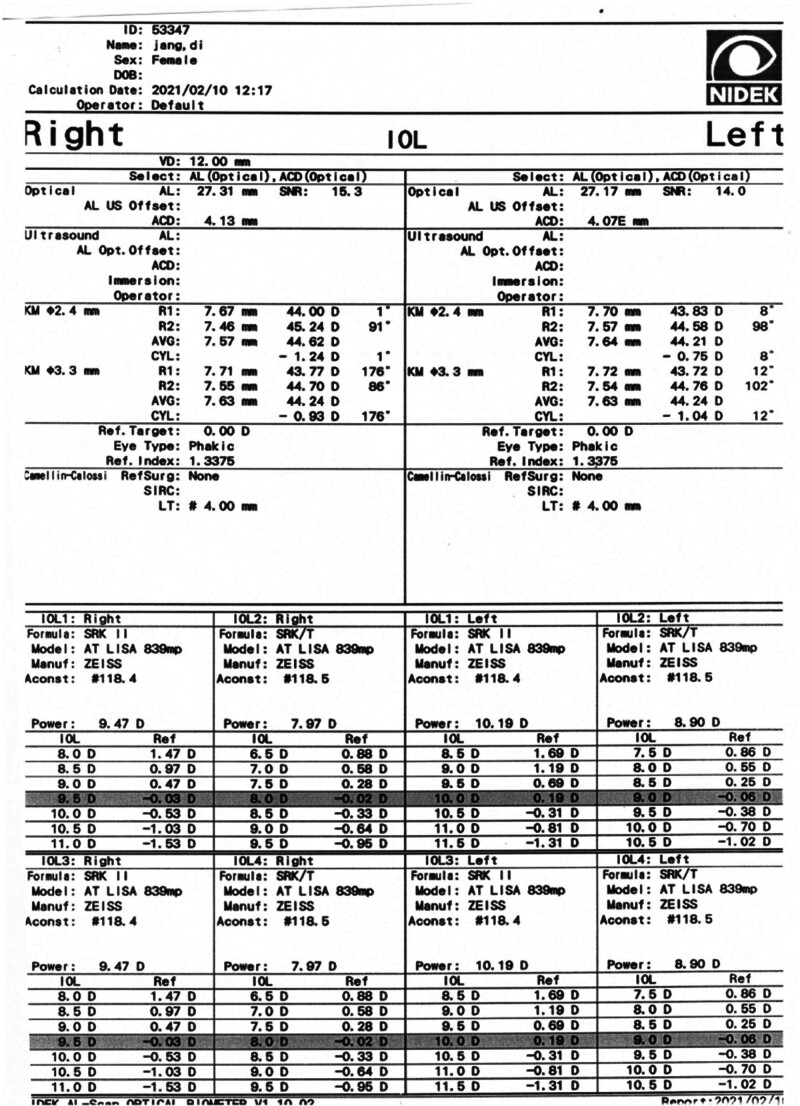
The Nidek AL Scan provides measurements of axial length, ACD, and keratometry. ACD = anterior chamber depth. This figure is used with the permission of ZEISS.

**Figure 4.**
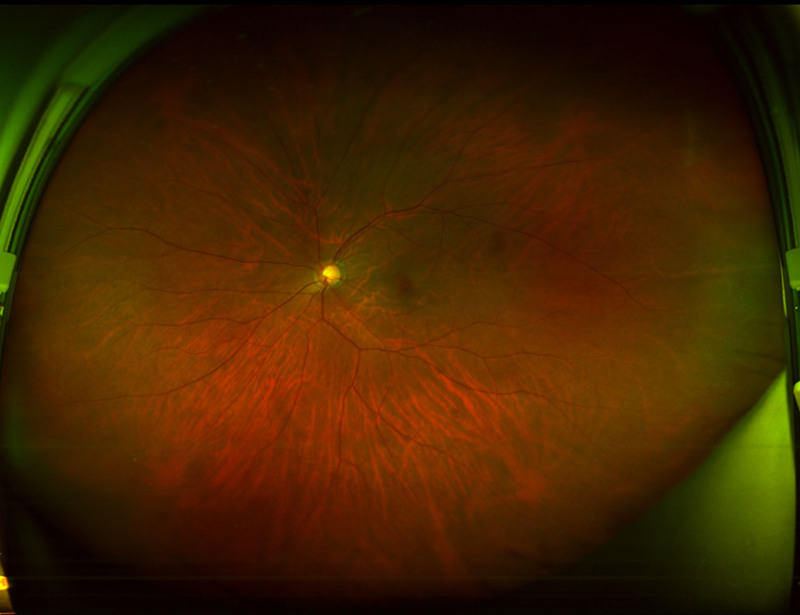
The wide-field fundus photography shows normal findings. This figure is used with the permission of ZEISS.

A 51-year-old female with high myopia (axial length: 27.17 mm) and a UDCA of 20/500 (CDVA corrected to 20/100) underwent phacoemulsification in her left eye on February 25, 2021 (Fig. 2). Preoperative autorefractor keratometer (ARK) readings showed −9.00 D sphere, −0.25 D cylinder at axis 10°, with keratometry values of K1: 43.75 D and K2: 44.25 D (Fig. 3). Intraocular pressure (IOP) was 14 mm Hg. The wide-field fundus photography using Optos did not reveal any abnormal findings (Fig. 4). A ZEISS LISA tri 839MP multifocal IOL was selected for implantation. The IOL was implanted using the BLUEMIXS 180 cartridge.

**Figure 5.**
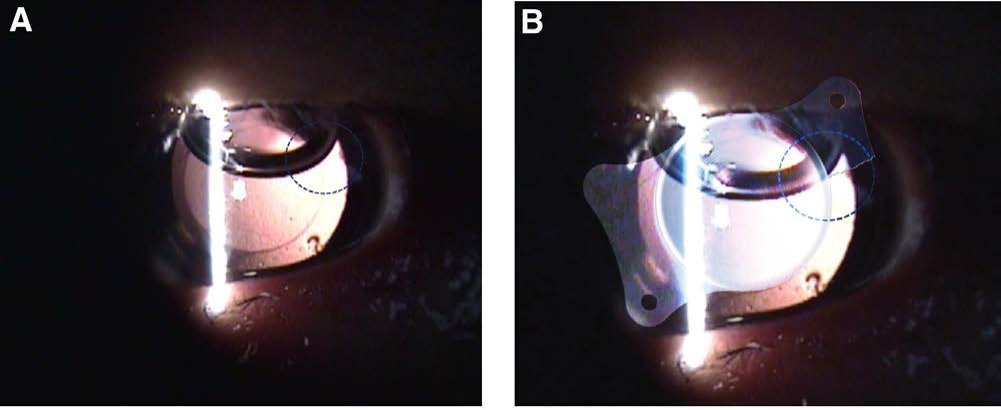
(A) The region marked at the 2 o’clock position with a blue dotted circle indicates the area where haptic loss occurred. (B) Since the damage was not clearly visible in the original image, an illustration of a torn haptic in the ZEISS LISA tri 839MP intraocular lens was created and digitally superimposed onto the image. The blue dotted circle highlights the location of the torn haptic. This figure is used with the permission of ZEISS.

During IOL insertion, 1 haptic became entangled on the injector tip and was torn. The damaged haptic was removed, and the IOL was positioned in the capsular bag, supported by the remaining 3 haptics (Fig. 5). After lens insertion, residual viscoelastic was removed using irrigation and aspiration. At the final stage, air was introduced into the anterior chamber to prevent collapse due to potential aqueous leakage, and surgery was completed without further complications. The patient was advised about potential future IOL decentration and the possibility of IOL exchange.

## 3. Postoperative course

On the first postoperative day, the patient’s uncorrected distance visual acuity (UDVA) improved to 20/20, with an IOP of 17 mm Hg and good IOL centration. The refraction was recorded as −1.00 D sphere, −1.25 D cylinder at an axis of 163°.

At the 2-week follow-up, UDVA slightly decreased to 20/22, while IOP remained stable at 17 mm Hg with continued good IOL centration. The refraction was recorded as −1.50 D sphere, −0.50 D cylinder at an axis of 15°.

At the 3-month follow-up, UDVA returned to 20/20, IOP remained stable at 15 mm Hg, and the IOL position was unchanged. The refraction was recorded as −0.75 D sphere, −1.00 D cylinder at an axis of 18°.

At the 6-month follow-up, UDVA was 20/22, with an IOP of 15 mm Hg and a well-centered IOL. The refraction measured −0.75 D sphere, −0.75 D cylinder at an axis of 15°.

At 7 months, a YAG laser posterior capsulotomy was performed, and anterior capsulotomy at the 12, 3, 6, and 9 o’clock positions was carried out to prevent capsular contraction. No signs of rhexis fibrosis were noted.

By the 1-year follow-up, UDVA remained at 20/22, with an IOP decrease to 13 mm Hg and stable IOL positioning. The refraction was −1.00 D sphere, −0.75 D cylinder at an axis of 13°.

**Figure 6.**
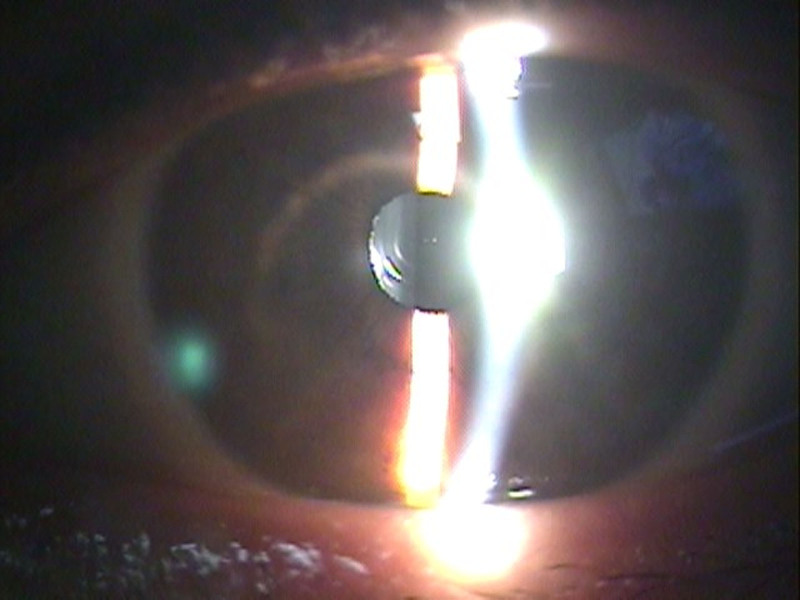
The centric ring is well-positioned within the pupil, indicating that the LISA tri lens is properly centered without pupil dilation. This figure is used with the permission of ZEISS.

At the final 3-year follow-up, UDVA improved again to 20/20, IOP was 14 mm Hg, and the IOL remained well-centered. The final refraction was −0.75 D sphere, −0.75 D cylinder at an axis of 14° (Fig. 6).

## 4. Discussion

This case demonstrates that the ZEISS LISA tri 839MP multifocal IOL can maintain long-term stability even in the absence of 1 haptic, particularly in highly myopic eyes where zonular support may be compromised.^[13]^ High myopia is associated with an increased risk of zonular dehiscence and IOL dislocation due to elongated axial length and thinner sclera.^[14,15]^ Given these risks, retaining the IOL rather than performing an exchange was a strategic decision to minimize additional stress on the zonules and reduce potential complications.^[16]^

While IOL exchange may sometimes be necessary, it carries inherent risks such as further zonular damage, posterior capsular rupture, anterior capsular tearing, increased inflammation, and prolonged recovery time. In contrast, preserving the existing IOL avoids these risks but necessitates careful long-term follow-up to ensure stability and optimal visual outcomes.

As axial length increases, the optic thickness of intraocular lenses (IOLs) tends to decrease due to the lower dioptric power required for myopic correction. However, the haptic thickness remains constant regardless of the IOL power. This structural characteristic may increase the likelihood of haptic damage during insertion, as the thinner optic makes the IOL more prone to instability within the injector. In high myopic patients, this instability can cause the haptic to get caught and torn as the IOL exits the injector.^[17]^ In this case, 1 haptic was torn during insertion, highlighting the importance of careful handling in high myopic eyes. Given the patient’s high myopia and associated zonular weakness, excessive manipulation during an IOL exchange could have further increased the risk of capsular rupture and destabilization. Additionally, modern multifocal IOLs are designed to provide adequate centration and stability even with minor structural compromises..

The YAG capsulotomy helped to avoid further decentration. Conservative management in such cases can be effective, as supported by recent studies. Limitations include the absence of pre- and postcapsulotomy images. The use of YAG laser capsulotomy was intended as a preventive measure against capsular contraction, a common complication in highly myopic patients, and to further stabilize the IOL. The favorable outcome observed over 3 years suggests that conservative management can be an effective strategy in similar cases, reducing the need for invasive procedures and maintaining a high quality of life for patients.^[18,19]^

This case supports existing literature that advocates for a cautious approach in cases of haptic damage, especially when IOL stability can be achieved with conservative management.^[20,21]^ While leaving a broken IOL in place is not the standard approach, this case demonstrates that in select situations, it can be a viable and safe alternative. The long-term stability seen here suggests that, in certain cases, a multifocal IOL with a missing haptic may continue to function effectively without necessitating immediate replacement.

## 5. Conclusion

In high myopic patients, a ZEISS LISA tri 839MP with a missing haptic segment can remain stable and provide excellent visual outcomes over long-term follow-up. Conservative management can be a viable option, reducing the need for IOL exchange and associated risks.

## Author contributions

**Conceptualization:** Jae-Ho Shin, Hui Jae Lee.

**Data curation:** Hui Jae Lee, Jong Won Lee, Jong Ho Lee.

**Investigation:** Hui Jae Lee.

**Methodology:** Hui Jae Lee.

**Software:** Jong Won Lee, Jong Ho Lee.

**Supervision:** Hui Jae Lee.

**Writing – original draft:** Hui Jae Lee.

**Writing – review & editing:** Hui Jae Lee.
